# STUDY METHODOLOGY AND SAMPLE SELECTION TO DEVELOP AGE-INCLUSIVE STRATEGIES

**DOI:** 10.1093/geroni/igad104.0642

**Published:** 2023-12-21

**Authors:** Yan Lin, Shu Xu

**Affiliations:** University of Massachusetts Boston, Boston, Massachusetts, United States; University of Massachusetts, Boston, Boston, Massachusetts, United States

## Abstract

Practical, feasible, and impactful age-friendly campus strategies to advance age inclusivity were identified through focus group and Delphi panel methodologies. Sixteen virtual focus groups with 78 participants (41 faculty, 16 students, and 21 staff/administrators; 44% ages 45-64 and 12% 65 and older) were conducted to assess age inclusivity in three institutional functions defined as high impact: teaching and learning, personnel, and student affairs. Facilitators presented participants with questions based on strategies and challenges identified from open-ended survey responses to our previous study. Following similar scripts for each of the three groups, facilitators asked 5 to 6 questions regarding potential strategies to address challenges such as those related to teaching older, age-diverse students; intergenerational tensions and biases; student support services; and incentives for faculty to integrate aging content into their courses. Next, the strategies yielded from this analysis were presented in online surveys to the Delphi panelists to assess their feasibility and impact. These 18 panelists had been selected based on their broad experience in higher education and upper-level administrative knowledge, professional connections, and/or expertise in diversity/inclusion/equity initiatives. In the first round, they evaluated the relative importance of 21 challenges to age inclusivity and their appropriateness, feasibility, and likelihood of implementation. The second-round survey narrowed the list of strategies to those rated as most viable (feasible and appropriate) in the first round and identified priorities among the selected strategies.

